# Prevalence and determinants of e-cigarette use among vocational college students: A cross-sectional study

**DOI:** 10.1371/journal.pone.0311585

**Published:** 2025-06-03

**Authors:** Siti Munisah Mohd Shoaib, Norliza Ahmad, Aidalina Mahmud

**Affiliations:** 1 Department of Community Health, Faculty of Medicine and Health Sciences, Universiti Putra Malaysia, Serdang, Selangor, Malaysia; 2 Ministry of Health, Putrajaya, Malaysia; Ministry of Health, General Health Directorate of Raparin and University of Raparin, IRAQ

## Abstract

**Introduction:**

E-cigarette use is rising globally, particularly among young adults, posing increasing health risks. This study examined the prevalence and factors associated with e-cigarette use among diploma students in a Malaysian vocational college.

**Methodology:**

A cross-sectional study was conducted among 700 students using probability proportionate to size sampling and a validated online questionnaire. Logistic regression identified factors associated with e-cigarette use, with p < 0.05 considered statistically significant.

**Results:**

The response rate was 87.7%, with an e-cigarette use prevalence of 29.0%. Significant factors included male gender (aOR = 5.2, 95% CI: 2.7–10.1), Sabah and Sarawak Bumiputera ethnicity(aOR = 83.1, 95% CI: 2.2–3146.3), perceived e-cigarette aids in quit smoking (aOR = 1.6, 95% CI: 1.2–2.1), perceived e-cigarette does not contain the toxic chemicals found in conventional cigarette (aOR = 1.4, 95% CI: 1.0–2.0), having close friends who use conventional cigarette (aOR = 2.1, 95% CI: 1.0–4.1) or e-cigarette (aOR = 8.0, 95% CI: 2.3–28.1), television exposure (aOR = 2.1, 95% CI: 1.0–4.2), positive attitude towards e-cigarette (aOR = 1.2, 95% CI: 1.1–1.2), and higher willingness (aOR = 1.2, 95% CI: 1.0–1.3) and intention (aOR = 1.4, 95% CI: 1.2–1.5) to use.

**Conclusion:**

E-cigarette use among students was influenced by gender, ethnicity, risk perceptions, peer influence, and media exposure. Targeted interventions addressing these factors are essential for reducing e-cigarette use in this population.

## Introduction

E-cigarettes are devices that heat a liquid, typically containing nicotine, flavorings, and other substances to produce an aerosol that is inhaled by the user. Their use poses significant health risks, particularly to young adults, youth, and pregnant women [1]. Growing concerns have emerged regarding the increasing prevalence of e-cigarette use. Previous studies among college and university students aged 18–26 have reported a wide range of prevalence rates. While no global prevalence of e-cigarette use is available, studies have reported varying rates among college students. In the United States, a study found a prevalence of 24.8%, while a study among university students in Northern Thailand reported a lower prevalence of 18.1%. Meanwhile, studies in Malaysia reported prevalence rates of 12.4% and 20.4% [2–5]. A study among vocational students in Thailand found an even higher prevalence of current e-cigarette use at 28.7%, with the highest percentage among those aged 20 years and older (30.8%) [6]. Although studies have examined e-cigarette use among adolescents in vocational schools, no research has specifically explored its use among young adults attending vocational colleges.

Nicotine in e-cigarette liquid may impair brain cognition and development, increase impulsive behavior, and negatively impact psychosocial health in young adults [7–9]. Other harmful chemicals in e-cigarettes, such as flavorings, diacetyl, propylene glycol (PG), carbon monoxide, formaldehyde, and polycyclic aromatic compounds, pose significant health risks [7,10]. In fact, respiratory problems, such as asthma, chronic obstructive pulmonary disease, eosinophilic pneumonia, epiglottitis, bronchitis, and acute respiratory distress, have been linked to e-cigarette use [7]. Additionally, e-cigarette or vaping use-associated lung injury (EVALI) has been reported among e-cigarette users, primarily linked to tetrahydrocannabinol (THC) and vitamin E acetate in certain e-cigarettes products [11,12].

Other harmful health risks of e-cigarette use include accidental nicotine overdose, increased risk of cardiopulmonary diseases, subsequent illicit drug use, serving as a gateway to conventional cigarettes, potentially leading to dual use of both types of cigarettes, and physical injuries from battery failures and explosions [7,13]. Small liquid droplets produced by e-cigarette aerosols have also been detected in the environment and on surfaces, potentially causing second-hand and third-hand exposure to people nearby [14,15]. Among those exposed to second-hand and third-hand e-cigarette aerosols, respiratory problems (especially among those with existing airway conditions such as asthma), DNA damage, immediate modifications in respiratory mechanics and exhaled inflammatory biomarkers, and an increased risk of cardiovascular disease have been observed [14,16–18].

One of the intrapersonal factors associated with e-cigarette use is having lower levels of knowledge about the potential negative health outcomes of e-cigarette use [19]. Additionally, poor perceptions of health risks and mental health have been observed among e-cigarette users [20–22]. E-cigarette use is also influenced by various external factors, including family members and peers, exposure to printed or online advertisements, the availability and affordability of e-cigarettes, positive attitudes towards e-cigarettes, higher intention and higher willingness to use, as well as engagement in high-risk behaviors have also been found to be associated with the use of e-cigarettes [6,20,21,23–30]. In Malaysia, the National Health Morbidity Survey (NHMS) 2019 indicated that the highest prevalence was among individuals aged 20–24 years (14.7%) [31]. However, studies among students at vocational colleges on e-cigarette use are scarce with no available data found among vocational college students in Malaysia. Hence, this study aimed to identify the prevalence and factors associated with e-cigarette use among young adults currently studying at a vocational college in Selangor, Malaysia.

## Materials and methods

### Study design and population

A cross-sectional study was conducted among diploma students at a vocational college in Selangor, Malaysia. Selangor, a state adjacent to Kuala Lumpur, the capital of Malaysia, was selected as the study site due to its high prevalence of e-cigarette use, as reported in the NHMS 2019. In addition, it houses the highest number of higher education institutions, with a total of 397,366 enrolments in 2022 [31,32]. Inclusion criteria included students currently enrolled in semester two or beyond, and young adults aged 18–26 years. Students who were on leave, not attending classes during the data collection period, or were suspended by the university were excluded. This study adhered to the Strengthening the Reporting of Observational Studies in Epidemiology (STROBE) guidelines for cross-sectional epidemiological studies in terms of design, setting, analysis, and reporting (S1 File STROBE checklist).

### Sample size and sampling

The required sample size was 700 participants, at 80% power, 95% confidence level, and 20% non-response rate. The required sample size was calculated using the two-population proportion formula (see S2 File Sample Size Calculation), based on the proportions of e-cigarette use across different monthly family income groups reported in a previous study [21]. Stratified random sampling with probability proportionate to size method was employed in this study. All 12 diploma programs (regarded as strata) in the selected college were included. Probability proportionate to size sampling was applied to each diploma course based on the calculated sample size. Finally, systematic random sampling was used to select eligible participants from each diploma course.

### Study instrument

This study used a set of validated questionnaires adapted from previous research [5,21,22,26–28,30,33,34]. The dependent variable of the study was e-cigarette use. The independent variables were sociodemographic characteristics, academic performance, reasons for using, knowledge of e-cigarette, health risk perception, mental health perception, family influence, peer influence, advertising media influences, availability, affordability, attitude towards e-cigarette use, willingness to use e-cigarette, intention to use e-cigarette, and high-risk behaviours.

It consisted of 12 sections. Part A of the questionnaire was regarding the sociodemographic characteristics and academic performance of the respondents. Part B of the questionnaire was regarding conventional cigarette and e-cigarette use profiles which were assessed by whether or not the respondent had smoked a conventional cigarette or use e-cigarette in the past 30 days with three response options [33]. E-cigarette users were then categorised into “never user” and “e-cigarette user” accordingly. Part C was regarding reasons for using e-cigarettes which were answered by those respondents who are occasional e-cigarette users and past month e-cigarette users only with six options were given [5]. The answer options for the questions will be “Yes” and “No”. Part D was regarding knowledge of e-cigarettes with eight statements [28]. The answer options were “Yes”, “No”, and “Don’t know”. Those who answered “Don’t know” were interpreted as wrong answers which were scored zero, while the correct answer was scored one. The minimum score was 0 and the maximum score was 8. A higher score will reflect good knowledge of e-cigarettes.

Part E consisted of two constructs on perceptions. The first construct consisted of five statements to assess health risk perceptions on e-cigarette [27]. It was scored using a 5-point Likert scale (1-totally disagree, 2-disagree, 3-unsure, 4-agree, 5-totally agree). The minimum score was 5 and the maximum score was 25. A lower score reflected that the respondents have a strong belief in the health risks of e-cigarettes. The second construct was on mental health perceptions [22]. It consisted of four questions on perceived anxiety, depression, loneliness, and stress symptoms during the past month. The questions were measured using a 5-point Likert scale (1-never, 2-rarely, 3-sometimes, 4-often, 5-always). The minimum score was 4 and the maximum score was 20. A higher score reflected poor mental health perceptions.

Part F was regarding social influences with two statements about behaviour of conventional cigarette and e-cigarette use among family members and another two statements about behaviour of conventional cigarette and e-cigarette use among close friend [27]. The answer option was “Yes” and “No”. Part G was regarding advertising media influences based on the exposure to e-cigarette advertisements [26]. A statement with six options was given with “Yes” and “No” answer options displayed for each option.

Part H was regarding e-cigarette availability and affordability [27]. The availability was explored by whether the respondents think it is difficult to get e-cigarettes. Meanwhile, the affordability was explored by whether the respondents think the price of e-cigarettes is affordable. The answer options for both questions were “Yes” and “No”. Part I, J and K was regarding attitude towards e-cigarettes, willingness to use, and intention to use respectively [34]. Attitude towards e-cigarette consisted of seven statements while willingness to use consisted of three statements and was scored using a 5-point Likert scale (1-totally disagree, 2-disagree, 3-unsure, 4-agree, 5-totally agree). Meanwhile, intention to use consisted of three questions and scored using a 5-point Likert scale (1-very unlikely, 2-unlikely, 3-unsure, 4-likely, 5-very likely). A higher score reflected a more positive attitude towards e-cigarettes, a higher willingness to use e-cigarettes, and a higher intention to use e-cigarettes. Part L was regarding high-risk behaviours with three statements on alcohol use, illegal drug use, and unprotected sex with single or multiple sexual partners [30]. The answer options were “Yes” and “No”. The complete questionnaire is available in the supplementary material (S3 File Questionnaire).

Pre-testing was conducted among 30 students from a diploma course to assess the stability of the questionnaire. Data from this pre-test was excluded from the final analysis. The internal consistency of the questionnaire was evaluated using Cronbach’s alpha, with values ranging from 0.70 to 0.96, indicating good to excellent reliability [35]. The Cronbach’s alpha values for each construct are summarized in Table 1.

**Table 1:** Summary of the Cronbach’s alpha index value.

Domain	Number of Items	Cronbach’s Alpha
Health risk perception	5	0.70
Mental health perceptions	6	0.77
Attitude towards e-cigarette	7	0.89
Willingness to use e-cigarette	3	0.74
Intention to use e-cigarette	3	0.96

### Data collection

Data was collected online using Google Forms, which were distributed to respondents through the WhatsApp application. Data collection took place from 29^th^ April to 17^th^ May 2024. Prior to data collection, a brief presentation detailing the study’s objectives and methodology was given to the head of the department. Respondent eligibility was assessed based on the inclusion and exclusion criteria.

### Statistical analysis

Data were analyzed using IBM SPSS version 29.0. Prior to analysis, the data sets were examined for out-of-range values and assessed for normality. Continuous independent variables were presented as mean ± standard deviation for normally distributed data and as median (interquartile range) for non-normally distributed data. Continuous independent variables, such as respondents’ age, monthly family income, and GPA, were categorized accordingly. Categorical variables were presented as frequencies and percentages. Bivariate analysis was conducted using the chi-square test for categorical independent variables and simple logistic regression for continuous independent variables to assess associations with e-cigarette use.

Results of bivariate analyses with p-value <0.25 were further tested for multicollinearity and interactions before being analyzed using multiple logistic regression to determine the factors associated with e-cigarette use [36]. Three regression methods were employed: the “Enter” method, the “Forward LR” method, and the “Backward LR” method. The most parsimonious model that best fits the data was selected. Results were reported as crude and adjusted odds ratios, with statistical significance set at p < 0.05.

### Efforts to minimize bias

Several measures were taken to reduce potential sources of bias. Probability proportionate to size sampling was used to ensure representative inclusion of students across different programs within the vocational college, minimizing selection bias. To reduce measurement bias, a validated and pre-tested online questionnaire was utilized. The survey was administered anonymously to encourage honest responses and reduce social desirability bias. Standardized definitions and clear wording of questions were applied consistently to limit information bias. Finally, logistic regression was used to adjust for potential confounding variables in the analysis.

### Ethics approval and consent to participate

Ethical clearance was obtained from the Ethics Committee for Research Involving Human Subjects, Universiti Putra Malaysia (JKEUPM-2024–193), in accordance with ethical standards. Approval from the Polytechnic and Community College Education Department, Ministry of Higher Education, was obtained prior to the pre-test and data collection. Online consent was obtained from all respondents via Google Forms. All information and details collected from the respondents were kept anonymous and transferred to a password-protected database linked to student identification created for this study. Access to this database was restricted to the investigators and will be maintained for seven years after the completion of the study, after which it will be deleted.

## Results

Out of 700 students invited to participate, 614 completed the survey, yielding a response rate of 87.7%. Non-participation (n = 86) was mainly due to 79 students not responding to the invitation and 7 students refusing to participate, despite receiving general consent information. No participants were excluded following survey submission. The participant flow is illustrated in the supplementary file (S4 File Flow diagram of study recruitment). The prevalence of exclusive e-cigarette use in this study was 29.0% (n = 178). Most respondents were aged between 18 and 20 years (73.0%), male (55.4%), of Malay ethnicity (87.6%), had a household average monthly income in the lower 40% (B40) income group (83.9%), and achieved a Grade Point Average (GPA) grade of B (76.2%). Table 2 presents the distribution of respondents according to sociodemographic characteristics and academic performance.

**Table 2:** Distribution of respondents according to sociodemographic characteristics and academic performance (n = 614).

Respondent characteristics	Mean±SD/ Median (IQR)	Frequency (n)	Percentage (%)
**Age**	20 (2)		
18–20 years old		448	73.0
21–25 years old		166	27.0
**Sex**			
Male		340	55.4
Female		274	44.6
**Race**			
Malay		538	87.6
Chinese		14	2.3
Indian		53	8.6
Others		9	1.5
**Monthly family income**	3000 (3000)		
B40		515	83.9
M40		78	12.7
T20		21	3.4
**GPA result**	3.21 ± 0.41		
A		93	15.1
B		468	76.2
C		53	8.7

The overall mean score for knowledge was 4.60 ± 1.64 out of 8. Respondents were almost equally distributed between those scoring 50% or below (45.3%) and those scoring above 50% (54.7%). Among the respondents, only 10 (1.6%) answered all the questions correctly, while 13 (2.1%) scored zero. Most respondents (88.6%) correctly identified that e-cigarettes represent a new form of addiction in society. Meanwhile, 75.2% incorrectly believed that e-cigarettes do not contain tar. Details on the distribution of knowledge among respondents are summarized in Table 3.

**Table 3:** Distribution of respondents according to knowledge of e-cigarettes (n = 614).

Knowledge of e-cigarette	Correct answer	Incorrect answer
	Frequency (%)	Frequency (%)
E-cigarettes do not harm the health of users	428 (69.7)	186 (30.3)
The liquid in e-cigarettes may contain nicotine	516 (84.0)	98 (16.0)
E-cigarettes do not contain tar	152 (24.8)	462 (75.2)
The sale of e-cigarettes has been banned throughout Malaysia	312 (50.8)	302 (49.2)
The use of e-cigarettes is one of a safe way to quit smoking	388 (63.2)	226 (36.8)
The liquid content in e-cigarettes has been adjusted by the Malaysian Ministry of Health	223 (36.3)	391 (63.7)
E-cigarette is a new form of addiction in society	544 (88.6)	70 (11.4)
The use of e-cigarettes has been declared illegal by the National Fatwa Council	260 (42.3)	354 (57.7)

Results of bivariate analyses identified eleven categorical independent variables and ten continuous independent variables with a p-value <0.25. Multiple logistic regression analysis using the “Forward LR” method revealed ten statistically significant factors: sex, race, perceiving that e-cigarettes aid in quitting smoking, perceiving that e-cigarettes do not contain toxic chemicals found in conventional cigarettes, e-cigarette and conventional cigarette usage by close friends, exposure to e-cigarette on television, attitude towards e-cigarettes, willingness to use, and intention to use (see Table 4). These determinants were consistent with the Hosmer and Lemeshow goodness-of-fit test (χ² = 11.049, df = 8, p = 0.199), which assesses how well the model fits the observed data. A non-significant p-value of >0.05 suggests that the model provides an adequate fit, meaning there is no significant difference between the predicted and actual outcomes. This model correctly classified 88.4% of e-cigarette use cases.

**Table 4:** Determinants of e-cigarette use.

Variables	Simple logistic regression		Multiple logistic regression					
	Crude OR	p-value	Coefficient	SE	Adjusted OR	95% CI		p-value
						Upper	Lower	
**Intercept**			−11.653					
**Age**								
18–20 years	Ref							
21–26 years	1.8	**0.002** ^*^						
**Sex**								
Female	Ref							
Male	7.3	**<0.001** ^*^	1.645	0.342	5.2	2.650	10.127	**<0.001** ^*^
**Race**								
Chinese	Ref							
Malay	5.5	0.102	1.161	1.473	3.2	0.178	57.340	0.430
Indian	3.4	0.252	1.385	1.549	4.0	0.192	83.213	0.371
Others	26.0	**0.009** ^*^	4.420	1.854	83.1	2.193	3146.339	**0.017** ^*^
**GPA result**								
A	Ref							
B	2.5	**0.003**						
C	7.4	**<0.001** ^*^						
**Knowledge of e-cigarette**	−0.2	**0.005** ^*^						
**Health risk perceptions**								
E-cigarettes help cut down the number of cigarettes smoked	0.3	**<0.001** ^*^						
E-cigarettes aid in quitting smoking	0.4	**<0.001** ^*^	0.459	0.146	1.6	1.189	2.108	**0.002** ^*^
E-cigarettes don’t contain toxic chemicals found in conventional cigarette	0.3	**0.002**	0.357	0.165	1.4	1.035	1.972	**0.030** ^*^
E-cigarettes are less harmful	0.3	**<0.001** ^*^						
E-cigarettes are less addictive	0.4	**<0.001** ^*^						
**Conventional cigarette usage by family members**								
No	Ref							
Yes	1.5	**0.017** ^*^						
**Conventional cigarette usage by close friends**								
No	Ref							
Yes	6.0	**<0.001** ^*^	0.721	0.346	2.1	1.043	4.053	**0.037** ^*^
**E-cigarette usage by close friends**								
No	Ref							
Yes	12.4	**<0.001** ^*^	2.088	0.636	8.1	2.320	28.072	**0.001** ^*^
**On television**								
No	Ref							
Yes	1.6	**0.030** ^*^	0.724	0.359	2.1	2.062	1.020	**0.044** ^*^
**Attitude towards e-cigarette**	0.2	**<0.001** ^*^	0.151	0.028	1.2	1.162	1.101	**<0.001** ^*^
**Willingness to use**	0.4	**<0.001** ^*^	0.163	0.062	1.2	1.177	1.041	**0.009** ^*^
**Intention to use**	0.4	**<0.001** ^*^	0.300	0.042	1.4	1.350	1.243	**<0.001** ^*^
**Alcohol use**								
No	Ref							
Yes	2.4	**0.007** ^*^						
**Illegal drugs use**								
No	Ref							
Yes	3.3	**0.021** ^*^						
**Unprotected sex**								
No	Ref							
Yes	4.3	**<0.001** ^*^						

Notes: Multiple logistic regression (Forward LR), no interaction (p > 0.05), no multicollinearity, Hosmer and Lemeshow goodness of fit test (p = 0.199), Omnibus model of coefficient (p < 0.001), Nagelkerke R^2^: 0.689

* Significant at p-value <0.05

The model indicates that male respondents were five times more likely to use e-cigarettes compared to females (aOR = 5.2, 95% CI: 2.7–10.1), while respondents from other races (Bumiputera Sabah and Sarawak) were 83 times more likely to use e-cigarettes compared to Chinese (aOR = 83.1, 95% CI: 2.2–3146.3). A one-unit increase in the perception that e-cigarettes aid in quitting smoking was associated with a 1.6-fold increase in the odds of e-cigarette use (aOR = 1.6, 95% CI: 1.2–2.1). Similarly, for each one-unit increase in the perception that e-cigarettes do not contain the toxic chemicals found in conventional cigarettes, the odds of e-cigarette use increased by 1.4 times (aOR = 1.4, 95% CI: 1.0–2.0).

Additionally, having close friends who smoke conventional cigarettes doubled the likelihood of e-cigarette use (aOR = 2.1, 95% CI: 1.0–4.1), while having close friends who use e-cigarettes increased the likelihood by eight times (aOR = 8.0, 95% CI: 2.3–28.1). Exposure to e-cigarette use on television was associated with a twofold increase in e-cigarette use (aOR = 2.1, 95% CI: 1.0–4.2). Moreover, a one-unit increase in positive attitudes towards e-cigarettes was associated with a 1.2-fold increase in the odds of e-cigarette use (aOR = 1.2, 95% CI: 1.1–1.2). Similarly, each one-unit increase in willingness to use and intention to use was linked to a 1.2-fold (aOR = 1.2, 95% CI: 1.0–1.3) and 1.4-fold (aOR = 1.4, 95% CI: 1.2–1.5) increase in the odds of e-cigarette use, respectively. Collectively, these variables accounted for 68.9% of the variance in e-cigarette use among diploma students. However, 31.1% of the variance remains unexplained, indicating the need for further investigation into additional influencing factors.

## Discussion

Our study analyzed e-cigarette use among diploma students at a vocational college in a state in Malaysia. The findings revealed that nearly one in three respondents were e-cigarette users, a prevalence notably higher than that reported in previous research among students in tertiary institutions. For instance, a study among university students in the U.S. aged 18–25 years reported an e-cigarette use prevalence of 24.8%, while local studies among university students in Malaysia reported prevalences of 12.4% and 20.4% [2–4]. In contrast, a study among students from 12 vocational institutions in Thailand reported a prevalence similar to our current study (28.7%) [6]. Overall, the high prevalence of e-cigarette use observed in this study raises significant concerns, particularly among vocational students.

Most respondents were generally aware that e-cigarettes are harmful. Besides nicotine, other chemicals such as diacetyl, PG, carbon monoxide, formaldehyde, and polycyclic aromatic compounds were found present in the e-cigarettes. These chemicals were also found to come along with heavy metals, aldehydes, and nicotine derivatives [7]. They were also aware that e-cigarettes represent a new form of addiction in society. However, more than half of the respondents were either unaware of or unsure about the content in e-cigarette liquids, for example whether it may contain nicotine and tar, which is consistent with previous study [28]. This highlights the critical need for enforcing greater transparency from manufacturers regarding product contents. Some e-cigarette liquids have been found to contain nicotine levels different from those indicated on the label [37]. Overall, respondents’ knowledge was rated as moderate, which is lower than the mean score of 5.80 ± 2.04 reported in a previous study [28]. Nevertheless, more than half of the respondents were aware of the potential health risks associated with e-cigarette use.

Regarding sex, previous studies have similarly reported a higher likelihood of e-cigarette use among males [21,30,38]. In many countries and cultures, including Malaysia, the use of e-cigarettes by males is more socially accepted, whereas females who use e-cigarettes often face cultural disapproval. Female users are frequently perceived as having low moral values and are subject to social ridicule [39]. Additionally, males were found to have lower health literacy, seek health information less frequently than females, and are more likely to perceive e-cigarettes as less harmful, making them more prone to use e-cigarettes and engage in other high-risk behaviors [13,40–42].

Our findings showed that individuals from other races, specifically the Bumiputera Sabah and Sarawak ethnic groups, were over 80 times more likely to use e-cigarettes compared to the Chinese population, while no significant association was observed for the Malay and Indian populations. However, this result should be interpreted with caution due to the small sample size of Chinese and other racial groups in our study, which may have influenced statistical significance. The higher prevalence of e-cigarette use among Bumiputera Sabah and Sarawak individuals may be attributed to several sociocultural factors. The National Health and Morbidity Survey (NHMS) 2019 reported that e-cigarette use was higher among Bumiputera Sabah (5.1%) and Bumiputera Sarawak (5.0%) compared to Chinese (3.5%) [31]. This disparity could reflect broader cultural and social norms surrounding smoking and tobacco alternatives within these communities. Studies have shown that indigenous groups in Malaysia, including Bumiputera Sabah and Sarawak, tend to have higher smoking rates [33], which may extend to e-cigarette use as a perceived safer alternative. In many rural and semi-urban communities in East Malaysia, tobacco use has deep historical and social roots, often associated with social bonding, masculinity, and traditional practices. Further qualitative studies are needed to explore these aspects. Given the sociocultural dynamics, interventions to reduce e-cigarette use among Bumiputera Sabah and Sarawak populations should be culturally tailored. Community-based health education programs, stricter regulatory measures on e-cigarette sales, and engagement with local leaders and influencers may be effective strategies to shift social norms and reduce e-cigarette uptake in these communities.

Regarding health risk perceptions, our findings are consistent with previous study in Jakarta which reported that health risk perceptions were associated with e-cigarette [27]. This misperception may arise from the early introduction of e-cigarettes as a supposedly safer alternative to smoking, along with aggressive marketing campaigns claiming their effectiveness in smoking cessation [13,43]. However, e-cigarettes are not a safer alternative, as their use has been linked to significant harm, and some users have become dual users of both e-cigarettes and conventional cigarettes [7,44].

Our findings on the belief that e-cigarettes do not contain the toxic chemicals found in conventional cigarettes are consistent with a previous study, which showed that a significantly higher percentage of e-cigarette users (15.5%) held this perception compared to non-users (7.3%) [27]. The e-cigarette market often promoted e-cigarettes as a healthier alternative to conventional cigarettes. However, recent findings indicate that the chemicals in e-cigarette liquids may pose health risks to both users and bystanders [15]. Additionally, there is a lack of transparency regarding the ingredients and chemicals composition of e-cigarette liquids, further complicating risk assessment [43,45].

Peer influence was found to be strongly associated with e-cigarette usage in this study. Specifically, the use of conventional cigarettes and e-cigarettes by close friends was found to be one of the factors in this study. Our findings align with previous research, which reported respondents who have friends who smoke conventional cigarettes and e-cigarette use were two times and ten times more likely to be associated with e-cigarette use [21,23]. This high level of association suggests that e-cigarettes might be increasingly viewed as a socially acceptable or preferred alternative to conventional cigarettes within peer groups. Peer influence significantly impacts individual behavior, especially among young adults, who are more likely to imitate the behaviors of their social circles [46,47]. An individual who is offered an e-cigarette by their friends is most likely to accept the offer, which may lead to continued use. Additionally, users may feel a sense of belonging within the e-cigarette community, reinforcing their behavior [48]. These behaviors can be explained by social learning, modeling, and the need for social identity and group belonging, which lower risk perception of e-cigarettes while increasing curiosity and experimentation.

Regarding exposure to e-cigarette use in mass media, such as television, our findings align with a previous U.S. study, which reported that young adults aged 18–24 who were exposed to e-cigarette advertisements on television had a significantly higher likelihood of e-cigarette use compared to other age groups [25]. Television, along with other print media, has been shown to receive the largest share of e-cigarette advertising [49]. However, these findings may vary by country, depending on the regulations governing e-cigarette advertising. In Malaysia, e-cigarettes are primarily advertised via print media and social media platforms [43,45]. The current trends in social media marketing and advertising should be carefully considered, especially since younger populations are among the highest users of these platforms. Exposure to e-cigarette marketing may contribute to the perception that e-cigarettes are harmless and non-addictive [49]. Media exposure creates a reinforcing cycle, where positive portrayals of e-cigarettes through marketing and social connections normalize their use. Effective regulation and counter-marketing strategies targeting these influences are essential to prevent e-cigarette initiation, particularly among youth.

Another factor identified in this study was attitude. Our finding is consistent with a study conducted in Thailand, which demonstrated that a positive attitude towards e-cigarettes was associated with e-cigarette usage [6]. Additionally, non-smokers were nearly three times more likely to have a negative attitude towards e-cigarettes compared to smokers [28]. An individual’s attitude towards e-cigarettes may be influenced by their knowledge, awareness, and health concerns. Attitudes are also shaped through observational learning and personal experience [50]. The significant findings observed in this study may be related to the moderate level of knowledge about e-cigarettes among respondents, which could influence their likelihood of e-cigarette use. This suggests the importance of addressing misconceptions about e-cigarettes and providing balanced information on e-cigarettes, as attitudes can be influenced by presenting new and accurate information.

Our finding on the willingness to use e-cigarettes aligns with previous research conducted among male high school students in Iran, which reported that a higher willingness to smoke cigarettes was associated with cigarette smoking [51]. Higher willingness to use e-cigarettes may be influenced by various factors, such as the appeal of flavors, particularly fruit flavors [52]. Other than that, decision-making processes in young adults tend to be more impulsive and less governed by long-term considerations. Compared to older adults, younger individuals may lack experience and fully developed judgement, which can influence their susceptibility to e-cigarette use [53].

Intention is one of the key components studied in the reasoned action pathway of behavioral models, where it has been consistently reported that higher intention is associated with a greater likelihood of performing the behavior. In line with this, this study found a significant association between intention to use and e-cigarette use. Our finding is consistent with a previous study conducted among university students in Saudi Arabia, which found that higher intention was significantly associated with e-cigarette use [54]. These findings highlighted the importance of understanding and addressing behavioral intentions in interventions targeting high-risk behaviors. By identifying the factors that influence individuals’ intentions to engage in such behaviors, prevention efforts can be tailored more effectively to promote healthier choices and reduce behaviors associated with adverse health outcomes.

The implications of this study call for greater efforts to reduce e-cigarette use among vocational college students. To our knowledge, this study provides one of the first detailed reports on multiple aspects of e-cigarette use within this population, where available data remains limited. Targeted interventions are urgently needed, with peer-led programs showing particular promise given the strong influence of peer behavior identified. Regulatory measures to limit exposure to e-cigarette advertising, especially on television and social media, are also essential. Furthermore, institutions should introduce “Quit Vaping” interventions alongside existing smoking cessation services, applying behavioral theories to address attitudes, willingness, and intention to use. As demonstrated by our findings, strengthening cessation support services across tertiary institutions may further enhance efforts to reduce e-cigarette use among young adults.

The strengths of this study include a satisfactory response rate of 87.7%, indicating a high level of participant engagement. This study is also the first to examine factors associated with e-cigarette use among students in a vocational college in Malaysia, addressing a significant gap in the literature. Another strength of this study is the sample’s demographic diversity, characterized by an almost equal gender distribution and involvement of students from all diploma courses. This diversity ensures that the findings are representative and generalizable to the broader population of students in tertiary vocational institutions. Additionally, this study provides valuable insights into e-cigarette use within this specific demographic, contributing to the limited research available on this topic in Malaysia.

This study has several limitations. First, its cross-sectional design limits the ability to infer causality between the identified factors and e-cigarette use. Second, data were self-reported, which may introduce recall bias and social desirability bias; however, the use of an anonymous online questionnaire likely reduced the latter. These biases could lead to underreporting of e-cigarette use, potentially underestimating its true prevalence. Third, although probability proportionate to size sampling was used to enhance representativeness, the study was conducted in a single vocational college, which may limit the generalizability of the findings to other student populations that differ in demographics, cultural context, or academic environment. Lastly, although efforts were made to minimize non-response, the 12.3% who did not participate could differ systematically from participants, possibly introducing non-response bias. The direction and magnitude of this bias are uncertain but may result in a slight underestimation or overestimation of associations, depending on the non-participants’ characteristics.

## Conclusion

This study found a high prevalence of e-cigarette use (29.0%) among diploma students in a Malaysian vocational tertiary institution, exceeding rates reported in earlier studies. Key factors associated with e-cigarette use included male gender, Bumiputera Sabah and Sarawak ethnicity, perceived reduced harm of e-cigarettes, peer influence, exposure to e-cigarette-related media (particularly television), positive attitudes, and greater willingness and intention to use. These findings highlight the need for targeted health education that addresses misconceptions about e-cigarettes and promotes cessation strategies. Future research should explore cultural and regional differences to better inform interventions. A comprehensive approach is essential to curb the rising trend of e-cigarette use among young adults in Malaysia.

## Supporting information

S1 File Strobe checklist. (DOCX)

S2 File Sample size calculation. (DOCX)

S3 File Questionnaire. (DOCX)

S4 File Flow diagram of study recruitment. (DOCX)

S5 File Study data. (XLSX)
