# Supplementary material for: Prevalence and determinants of e-cigarette use among vocational college students: A cross-sectional study
Source: PLoS One. 2025 Jun 3;20(6):e0311585. doi: 10.1371/journal.pone.0311585 (PMC12132976; doi:10.1371/journal.pone.0311585)
Supplement: S1 File — (DOCX) [file pone.0311585.s001.docx]

STROBE Statement—Checklist of items that should be included in reports of ***cross-sectional studies***

|  | Item No | Recommendation | Subheading of article |
| --- | --- | --- | --- |
| **Title and abstract** | 1 | (*a*) Indicate the study’s design with a commonly used term in the title or the abstract  *Prevalence and determinants of e-cigarette use among vocational college students: A cross-sectional study* | Title |
|  |  | (*b*) Provide in the abstract an informative and balanced summary of what was done and what was found  *Abstract in this study consists of introduction, methodology, results, and conclusion sections with informative and balanced information* | Abstract |
| Introduction | | | |
| Background / rationale | 2 | Explain the scientific background and rationale for the investigation being reported  *We provided specific background related to the limited data on prevalence of e-cigarette use among vocational college students, the health risks of e-cigarette use in public health. We stated in the section: “Growing concerns have emerged regarding the increasing prevalence of e-cigarette use. Previous studies among college and university students aged 18 to 26 have reported a wide range of prevalence rates. While no global prevalence of e-cigarette use is available, studies have reported varying rates among college students. Furthermore, the usage of e-cigarette exposed them to various health implications due to presence of nicotine and other harmful chemicals such as flavorings, diacetyl, propylene glycol (PG), carbon monoxide, formaldehyde, and polycyclic aromatic compounds.” Due to this gap, the study was conducted.* | Introduction |
| Objectives | 3 | State specific objectives, including any prespecified hypotheses  *Hence, this study aimed to identify the prevalence and factors associated with e-cigarette use among young adults currently studying at a vocational college in Selangor, Malaysia.* | Introduction |
| Methods | | | |
| Study design | 4 | Present key elements of the study design early in the paper  *This study was a cross-sectional study. “A cross-sectional study was conducted among diploma students at a vocational college in Selangor, Malaysia.”* | Study design and population |
| Setting | 5 | Describe the setting, locations, and relevant dates, including periods of recruitment, exposure, follow-up, and data collection  *Setting of study: A diploma student at a vocational college in Selangor, Malaysia, who fulfilled the inclusion and exclusion criteria, was included in the study.*  *Locations of study: “Selangor, a state adjacent to Kuala Lumpur, the capital of Malaysia, was selected as the study site due to its high prevalence of e-cigarette use, as reported in the NHMS 2019. In addition, it houses the highest number of higher education institutions, with a total of 397,366 enrolments in 2022 (31,32).”*  *Relevant dates of study or data collection: “Data collection took place from 29th April to 17th May 2024.”* | Study design and population  Data collection |
| Participants | 6 | (*a*) Give the eligibility criteria, and the sources and methods of selection of participants  *Eligible criteria in this study: “Inclusion criteria included students currently enrolled in semester two or beyond, and young adults aged 18 to 26 years. Students who were on leave, not attending classes during the data collection period, or were suspended by the university were excluded.”*  *Stratified random sampling with probability proportionate to size method was employed in this study. All 12 diploma programs (regarded as strata) in the selected college were included. Probability proportionate to size sampling was applied to each diploma course based on the calculated sample size. Systematic random sampling was used to select eligible participants from each diploma course.* | Study design and population |
| Variables | 7 | Clearly define all outcomes, exposures, predictors, potential confounders, and effect modifiers. Give diagnostic criteria, if applicable  *The dependent variable of the study was e-cigarette use. The dependent variables were sociodemographic characteristics, academic performance, reasons for using, knowledge of e-cigarette, health risk perception, mental health perception, family influence, peer influence, advertising media influences, availability, affordability, attitude towards e-cigarette use, willingness to use e-cigarette, intention to use e-cigarette, and high-risk behaviours.* | Study instrument |
| Data sources/ measurement | 8* | For each variable of interest, give sources of data and details of methods of assessment (measurement). Describe comparability of assessment methods if there is more than one group  *Sources of questionnaire: “This study used a set of validated questionnaires adapted from previous research (5,21,22,26-28,30,33,34).”*  *Interest variables (included dependent and independent variables) were assessed by questionnaire through Google form. “Data was collected online using Google Forms, which were distributed to respondents through the WhatsApp application.”*  *In this study, all respondents were the students from the same vocational college and the same method was used to assess variables of interest in all respondents* | Study instrument  Data collection |
| Bias | 9 | Describe any efforts to address potential sources of bias  *Efforts to address potential source of bias: To minimize selection bias, probability proportionate to size sampling was employed to ensure representativeness across different student groups within the college. A validated questionnaire was used to reduce measurement bias, and anonymity was maintained to encourage honest responses and reduce social desirability bias. Additionally, clear definitions and standardized questions were used to minimize information bias* *and logistic regression was applied to adjust for potential confounders in the analysis.* | Efforts to minimize bias |
| Study size | 10 | Explain how the study size was arrived at  *In this study, study size refers to sample size. “The sample size was determined using the two-population proportion formula, based on e-cigarette use and monthly family income from a previous study (21).”* | Sampling size and sampling |
| Quantitative variables | 11 | Explain how quantitative variables were handled in the analyses. If applicable, describe which groupings were chosen and why  *Quantitative variables were categorized to facilitate analysis and comparison. The dependent variable, e-cigarette use, was dichotomized into 'never users' and 'e-cigarette users' based on participants’ responses. Among the independent variables, age was categorized using the sample's median age, while monthly family income was classified according to Malaysia’s official income group thresholds (as of April 2024). These categorizations allowed for clearer interpretation of associations and ensured comparability with findings from previous studies.* | Study instrument and statistical analysis |
| Statistical methods | 12 | (*a*) Describe all statistical methods, including those used to control for confounding  *“Bivariate analysis was conducted using the chi-square test for categorical independent variables and simple logistic regression for continuous independent variables to assess associations with e-cigarette use. Results of bivariate analyses with p-value <0.25 were further tested for multicollinearity and interactions before being analyzed using multiple logistic regression to determine the factors associated with e-cigarette use. Results were reported as crude and adjusted odds ratios, with statistical significance set at p < 0.05.”* | Statistical analysis |
|  |  | (*b*) Describe any methods used to examine subgroups and interactions  *Interaction were checked using logistic regression before the variables were proceed to be analysed using multiple logistic regression.* | Statistical analysis |
|  |  | (*c*) Explain how missing data were addressed  *There was no missing data as Google form ‘required’ the participants to answer all of the question to complete the questionnaire* |  |
|  |  | (*d*) If applicable, describe analytical methods taking account of sampling strategy  *Probability proportionate to size (PPS) sampling was used to ensure proportional representation of students from different programs within the vocational college. During analysis, this sampling strategy was accounted for by applying logistic regression models using the collected data without weighting, as the sample proportions reflected the actual population structure. The analysis focused on identifying associations while preserving the representativeness provided by the PPS method.* | Statistical analysis |
|  |  | (*e*) Describe any sensitivity analyses  *No sensitivity analyses were conducted, as there were no missing data and the primary model was based on pre-specified variable categorizations.* |  |
| Results | | | |
| Participants | 13* | (a) Report numbers of individuals at each stage of study—eg numbers potentially eligible, examined for eligibility, confirmed eligible, included in the study, completing follow-up, and analysed  *“Out of 700 randomly selected respondents invited from the sampling list, 614 students participated, resulting in an overall response rate of 87.7%.”* | Results |
|  |  | (b) Give reasons for non-participation at each stage  *Of the 700 students invited to participate, 614 completed the survey, resulting in a response rate of 87.7%. Non-participation (n = 86) was primarily due to absence during data collection or refusal to participate, despite providing general consent information. No participants were excluded after the survey submission.* | Results |
|  |  | (c) Consider use of a flow diagram  Flow diagram as in supplementary file. | S4 Flow diagram of study recruitment |
| Descriptive data | 14* | (a) Give characteristics of study participants (eg demographic, clinical, social) and information on exposures and potential confounders  *The characteristics of study participants are summarized in Table 2.* | Results |
|  |  | (b) Indicate number of participants with missing data for each variable of interest  *In this study, there was no participant with missing data.* |  |
| Outcome data | 15* | Report numbers of outcome events or summary measures  *“The prevalence of exclusive e-cigarette use in this study was 29.0% (n = 178).”* | Results |
| Main results | 16 | (*a*) Give unadjusted estimates and, if applicable, confounder-adjusted estimates and their precision (eg, 95% confidence interval). Make clear which confounders were adjusted for and why they were included  *In this study, unadjusted estimates (univariate analysis) and adjusted estimates are calculated for dependent variable and independent variable and both of them provided in Table 4.* | Results |
|  |  | (*b*) Report category boundaries when continuous variables were categorized  *Independent variables, age were grouped based on median age, monthly family income was grouped following Malaysia’s income group threshold (as of April 2024).* | Study instrument |
|  |  | (*c*) If relevant, consider translating estimates of relative risk into absolute risk for a meaningful time period  *Not applicable.* |  |
| Other analyses | 17 | Report other analyses done—eg analyses of subgroups and interactions, and sensitivity analyses  *Not applicable.* |  |
| Discussion | | | |
| Key results | 18 | Summarise key results with reference to study objectives  *The key findings are explained throughout the discussion section with comparison with other studies.* | Discussion |
| Limitations | 19 | Discuss limitations of the study, taking into account sources of potential bias or imprecision. Discuss both direction and magnitude of any potential bias  *Here we discussed the limitation of our study. “This study has several limitations. First, its cross-sectional design limits the ability to infer causality between the identified factors and e-cigarette use. Second, data were self-reported, which may introduce recall bias and social desirability bias; however, the use of an anonymous online questionnaire likely reduced the latter. These biases could lead to underreporting of e-cigarette use, potentially underestimating its true prevalence. Third, although probability proportionate to size sampling was used to enhance representativeness, the study was conducted in a single vocational college, which may limit the generalizability of the findings to other student populations that differ in demographics, cultural context, or academic environment. Lastly, although efforts were made to minimize non-response, the 12.3% who did not participate could differ systematically from participants, possibly introducing non-response bias. The direction and magnitude of this bias are uncertain but may result in a slight underestimation or overestimation of associations, depending on the non-participants' characteristics.”* | Discussion |
| Interpretation | 20 | Give a cautious overall interpretation of results considering objectives, limitations, multiplicity of analyses, results from similar studies, and other relevant evidence  *Some cautious are given in the discussion related to our findings and our proposed approaches in tackling high prevalence of e-cigarette use among vocational college students. Then we also mentioned the limitations of our study in the strength and limitation of the study.* | Discussion |
| Generalisability | 21 | Discuss the generalisability (external validity) of the study results  *It has been included as one of the limitations of the study.* | Discussion |
| Other information | | | |
| Funding | 22 | Give the source of funding and the role of the funders for the present study and, if applicable, for the original study on which the present article is based  *Not applicable.* |  |

*Give information separately for exposed and unexposed groups.

**Note:** An Explanation and Elaboration article discusses each checklist item and gives methodological background and published examples of transparent reporting. The STROBE checklist is best used in conjunction with this article (freely available on the Web sites of PLoS Medicine at http://www.plosmedicine.org/, Annals of Internal Medicine at http://www.annals.org/, and Epidemiology at http://www.epidem.com/). Information on the STROBE Initiative is available at www.strobe-statement.org.
