# Supplementary material for: Prevalence and determinants of e-cigarette use among vocational college students: A cross-sectional study
Source: PLoS One. 2025 Jun 3;20(6):e0311585. doi: 10.1371/journal.pone.0311585 (PMC12132976; doi:10.1371/journal.pone.0311585)
Supplement: S2 File — (DOCX) [file pone.0311585.s002.docx]

## **Sampling Size Estimation**

The sample size for the study to determine prevalence is calculated using the formula below:

$$n= \frac{Z^{2}P\left( 1-P \right)}{d^{2}}$$

where,

n = Sample size

Z = Z statistic for a level of confidence (1.96 for 95% confidence level)

P = Expected prevalence or proportion

d = Precision (5%)

The selected sample size to determine prevalence was based on the prevalence of current e-cigarette use among university students in selected university in Klang Valley, Malaysia done by Wan Puteh et al. (2018).

P = 20.4% = 0.204 (Proportion of e-cigarette use among university students)

(Wan Puteh et al., 2018)

$$n= \frac{{1.96}^{2}0.204\left( 1-0.204 \right)}{{0.05}^{2}}$$

= 250

The sample size (n) in this study was calculated using the two population proportions formula from Lwanga & Lemeshow (1991). The formula is shown below:

$$n= \frac{\left[ z_{1-\frac{\alpha}{2}}\sqrt{2\bar{P}\left( 1-\bar{P} \right)}+z_{1-\beta}\sqrt{P_{1}\left( 1-P_{1} \right)+P_{2}\left( 1-P_{2} \right)} \right]^{2}}{\left( P_{1}- P_{2} \right)^{2}}$$

where,

n = sample size

$z_{1-\frac{\alpha}{2}}$ = standard error associated with 95% confidence interval = 1.96

$z_{1-\beta}$ = standard error associated with 80% power = 0.842

P_1_ = population proportion 1

P_2_ = population proportion 2

$\bar{P}$ = $\frac{P_{1}+P_{2}}{2}$

The estimation of the sample size in this study was based on the monthly family income and e-cigarette use, as this study measured monthly family income as one of the independent variables and e-cigarette use as the dependent variable. The calculation below was based on a cross-sectional study of personal and perceptual factors associated with the use of e-cigarettes among university students in northern Thailand (Phetphum et al., 2021).

P1 = 13.9% = 0.139 (Proportion of monthly income lower than mean)

(Phetphum et al., 2021)

P2 = 22.9% = 0.229 (Proportion of monthly income higher than mean)

(Phetphum et al., 2021)

$\bar{P}$ = (0.139+0.229)/2 = 0.184

$$n= \frac{\left[ 1.96\sqrt{0.368\left( 1-0.184 \right)}+0.842\sqrt{0.139\left( 1-0.139 \right)+0.229\left( 1-0.229 \right)} \right]^{2}}{\left( 0.139- 0.229 \right)^{2}}$$

= 290

Taking into account adjustment for comparison between two groups

= 290 x 2

= 580

Taking into account adjustment for non-response of 20%

= 580 + [(0.2) (580)]

**= 696 final sample size**

The sample size estimation which is 696 which was rounded up to 700 was chosen for this study.
