# Supplementary material for: Prevalence and determinants of e-cigarette use among vocational college students: A cross-sectional study
Source: PLoS One. 2025 Jun 3;20(6):e0311585. doi: 10.1371/journal.pone.0311585 (PMC12132976; doi:10.1371/journal.pone.0311585)
Supplement: S3 File — (DOCX) [file pone.0311585.s003.docx]

| Nombor rujukan / *Reference number:* |  |  |  |  |
| --- | --- | --- | --- | --- |

**
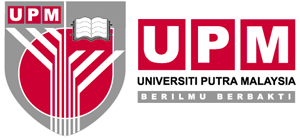
**

**Fakulti Perubatan dan Sains Kesihatan**

***Faculty of Medicine and Health Sciences***

**Jabatan Kesihatan Komuniti**

***Community Health Department***

**Soal Selidik**

***Questionnaire***

**Tajuk Kajian:**

***Study Title:***

**Taburan dan Faktor yang Menyumbang Kepada Penggunaan Rokok Elektronik Dalam Kalangan Pelajar Yang Menjalani Kursus Diploma di Politeknik Sultan Salahuddin Abdul Aziz Shah (PSA), Shah Alam, Selangor**

***Prevalence and Associated Factors of Electronic Cigarette Among Diploma Students in Politeknik Sultan Salahuddin Abdul Aziz Shah (PSA), Shah Alam, Selangor***

# **SOAL SELIDIK / *QUESTIONAIRE***

## **Bahagian A : Ciri-ciri Sosiodemografi & Prestasi Akademik**

***Part A : Sociodemographic Characteristics & Academic Performance***

Isikan tempat kosong atau tandakan ( / ) pada kotak yang disediakan. Sila jawab semua soalan.

*Fill in the blanks or tick ( / ) in the boxes provided. Please answer all questions.*

| 1. | Umur  *Age* |  | **:** | **_____** tahun / *years old* |
| --- | --- | --- | --- | --- |

| 2. | Jantina | : | Lelaki / *Male* |  |
| --- | --- | --- | --- | --- |
|  | *Sex* |  | Perempuan / *Female* |  |

| 3. | Bangsa | : | Melayu / *Malay* |  |
| --- | --- | --- | --- | --- |
|  | *Race* |  | Cina / *Chinese* |  |
|  |  |  | India / *Indian* |  |
|  |  |  | Lain-lain / *Others* |  |

| 4. | Pendapatan bulanan keluarga | : | RM |  |
| --- | --- | --- | --- | --- |
|  | *Monthly family income* |  |  |  |

| 5. | Keputusan GPA (Semester terakhir) | : | ________ |
| --- | --- | --- | --- |
|  | *GPA result (Latest semester)* |  |  |

## **Bahagian B: Penggunaan Rokok Tembakau dan Rokok Elektronik**

***Part B : Conventional Cigarette and E-cigarette Use***

Soalan-soalan berikut memerlukan jawapan “Ya” atau “Tidak”. Sila tandakan ( / ) pada kotak untuk mewakili jawapan anda. Sila jawab semua soalan.

*The following questions require “Yes” or “No” answers. Please ( / ) in the boxes representing your answer. Please answer all questions.*

**1. Dalam tempoh 30 hari yang lepas, adakah anda merokok rokok tembakau? *During the past 30 days, did you smoke conventional cigarette?***

| **No. / *Num.*** | **Pernyataan / *Statement*** | **Ya / *Yes*** | **Tidak / *No*** |
| --- | --- | --- | --- |
| a. | Tidak, saya tidak pernah merokok rokok tembakau / *No, I have never smoked conventional cigarette* |  |  |
| b. | Tidak, tetapi saya pernah merokok rokok tembakau sebelum 30 hari yang lepas / *No, but I have smoked conventional cigarette before the past 30 days* |  |  |
| c. | Ya / *Yes* |  |  |

**2. Dalam tempoh 30 hari yang lepas, adakah anda menggunakan rokok elektronik?**

***During the past 30 days, did you use e-cigarette?***

| **No. / *Num.*** | **Pernyataan / *Statement*** | **Ya / *Yes*** | **Tidak / *No*** |
| --- | --- | --- | --- |
| a. | Tidak, saya tidak pernah menggunakan rokok elektronik / *No, I have never used e-cigarette* |  |  |
| b. | Tidak, tetapi saya pernah menggunakan rokok elektronik sebelum 30 hari yang lepas */ No, but I have used e-cigarette before the past 30 days* |  |  |
| c. | Ya / *Yes* |  |  |

Arahan: Jika anda tidak pernah menggunakan rokok elektronik, sila langkau Bahagian C dan teruskan menjawab Bahagian D dan seterusnya.

*Instruction: If you have never used e-cigarette, please skip Part C and proceed to Part D and the next.*

## **Bahagian C: Alasan untuk Menggunakan Rokok Elektronik**

***Part C : Reasons to Use E-cigarette***

Soalan-soalan berikut memerlukan jawapan “Ya” atau “Tidak”. Sila tandakan ( / ) pada kotak untuk mewakili jawapan anda. Sila jawab semua soalan.

*The following questions require “Yes” or “No” answers. Please ( / ) in the boxes representing your answer. Please answer all questions.*

| **No. / *Num.*** | **Pernyataan / *Statement*** | **Ya / *Yes*** | **Tidak / *No*** |
| --- | --- | --- | --- |
| 1. | Keinginan diri sendiri / *Own desire* |  |  |
| 2. | Pengaruh sosial (Keluarga atau rakan) / *Social influence (Family or friends)* |  |  |
| 3. | Kepercayaan peribadi / *Personal belief* |  |  |
| 4. | Trend semasa / *Current trends* |  |  |
| 5. | Untuk berhenti merokok / *Aim to quit smoking* |  |  |
| 6. | Emosi diri sendiri (bosan, kesunyian, stres) / *Self-emotion (boredom, loneliness, stress)* |  |  |

## **Bahagian D : Pengetahuan Mengenai Rokok Elektronik**

***Part D : Knowledge of E-cigarette***

Soalan-soalan berikut memerlukan jawapan “Ya”, “Tidak” atau “Tidak tahu”. Sila tandakan ( / ) pada kotak untuk mewakili jawapan anda. Sila jawab semua soalan.

*The following questions require “Yes”, “No” or “Don’t know” answers. Please ( / ) in the boxes representing your answer. Please answer all questions.*

| **No. / Num.** | **Pernyataan / Statement** | **Ya / Yes** | **Tidak / No** | **Tidak tahu / Don’t know** |
| --- | --- | --- | --- | --- |
| 1. | Rokok elektronik tidak membahayakan kesihatan pengguna / *E-cigarette do not harm the health of users* |  |  |  |
| 2. | Cecair di dalam rokok elektronik mungkin mengandungi nikotin / *The liquid in e-cigarette may contains nicotine* |  |  |  |
| 3. | Rokok eletronik tidak mengandungi tar / *E-cigarette does not contain tar* |  |  |  |
| 4. | Penjualan rokok elektronik telah diharamkan di seluruh Malaysia / *The sale of e-cigarette has been banned throughout Malaysia* |  |  |  |
| 5. | Penggunaan rokok elektronik merupakan salah satu cara yang selamat untuk berhenti merokok / *The use of e-cigarette is one of safe way to quit smoking* |  |  |  |
| 6. | Kandungan cecair di dalam rokok elektronik telah diselaraskan oleh Kementerian Kesihatan Malaysia / *The liquid content in e-cigarette has been adjusted by the Malaysian Ministry of Health* |  |  |  |
| 7. | Rokok elektronik adalah satu alat ketagihan baru dalam kalangan masyarakat / *E-cigarette is a new form of addiction in society* |  |  |  |
| 8. | Penggunaan rokok elektronik telah diisytiharkan haram oleh Majlis Fatwa Kebangsaan / *The use of e-cigarette has been declared illegal by the National Fatwa Council* |  |  |  |

## **Bahagian E : Persepsi**

***Part E : Perceptions***

**a. Persepsi Risiko Kesihatan / *Health Risk Perception***

Sila tandakan ( / ) mengikut tahap persetujuan anda terhadap pernyataan di bawah mengikut skala berikut. Sila jawab semua soalan.

*Please tick ( / ) on how much you agree with the statement based on the following scale. Please answer all questions.*

| **1** | **2** | **3** | **4** | **5** |
| --- | --- | --- | --- | --- |
| Sangat tidak setuju / *Totally disagree* | Tidak setuju / *Disagree* | Tidak pasti / *Unsure* | Setuju / *Agree* | Sangat setuju / *Totally agree* |

| **No. / *Num.*** | **Pernyataan / *Statement*** | **1** | **2** | **3** | **4** | **5** |
| --- | --- | --- | --- | --- | --- | --- |
| 1. | Rokok elektronik membantu mengurangkan jumlah rokok tembakau yang dihisap / *E-cigarette help cut down the number of cigarette smoked.* |  |  |  |  |  |
| 2. | Rokok elektronik membantu untuk berhenti merokok rokok tembakau / *E-cigarette aids in quitting cigarette smoking* |  |  |  |  |  |
| 3. | Rokok elektronik tidak mengandungi bahan kimia beracun yang terdapat dalam rokok tembakau / *E-cigarette doesn’t contain toxic chemicals that have been found in conventional cigarette* |  |  |  |  |  |
| 4. | Rokok elektronik adalah kurang berbahaya / *E-cigarette is less harmful* |  |  |  |  |  |
| 5. | Rokok elektronik kurang menyebabkan ketagihan / *E-cigarette is less addictive* |  |  |  |  |  |

**b. Persepsi Kesihatan Mental / *Mental Health Perceptions***

Sila tandakan ( / ) mengikut tahap persetujuan anda terhadap pernyataan di bawah mengikut skala berikut. Sila jawab semua soalan.

*Please tick ( / ) on how much you agree with the statement based on the following scale. Please answer all questions.*

| **1** | **2** | **3** | **4** | **5** |
| --- | --- | --- | --- | --- |
| Tidak pernah / *Never* | Jarang / *Rarely* | Kadang-kadang / *Sometimes* | Selalu / *Often* | Sentiasa / *Always* |

| **No. / *Num.*** | **Pernyataan / *Statement*** | **1** | **2** | **3** | **4** | **5** |
| --- | --- | --- | --- | --- | --- | --- |
| 1. | Sepanjang bulan lalu, berapa kerapkah anda berasa cemas? / *During the past month, how often have you felt anxious?* |  |  |  |  |  |
| 2. | Sepanjang bulan lalu, berapa kerapkah anda berasa tertekan (murung)? / *During the past month, how often have you felt depressed?* |  |  |  |  |  |
| 3. | Sepanjang bulan lalu, berapa kerapkah anda berasa sunyi? / *During the past month, how often have you felt lonely?* |  |  |  |  |  |
| 4. | Sepanjang bulan lalu, berapa kerapkah anda berasa stres? / *During the past month, how often have you felt stressed?* |  |  |  |  |  |

## **Bahagian F : Pengaruh Sosial**

***Part F : Social Influences***

Soalan-soalan berikut memerlukan jawapan “Ya” atau “Tidak”. Sila tandakan ( / ) pada kotak untuk mewakili jawapan anda. Sila jawab semua soalan.

*The following questions require “Yes” or “No” answers. Please ( / ) in the boxes representing your answer. Please answer all questions.*

| **No. / *Num.*** | **Pernyataan / *Statement*** | **Ya / *Yes*** | **Tidak / *No*** |
| --- | --- | --- | --- |
| 1. | Saya mempunyai ahli keluarga yang menggunakan rokok tembakau / *I have family members who smoke conventional cigarette* |  |  |
| 2. | Saya mempunyai ahli keluarga yang menggunakan rokok elektronik / *I have family members who use e-cigarette* |  |  |
| 3. | Saya mempunyai kawan rapat yang menggunakan rokok tembakau / *I have close friends who smoke conventional cigarette* |  |  |
| 4. | Saya mempunyai kawan rapat yang menggunakan rokok elektronik / *I have close friends who use e-cigarette* |  |  |

## **Bahagian G : Pengaruh Media Pengiklanan**

***Part G : Advertising Media Influences***

Soalan-soalan berikut memerlukan jawapan “Ya” atau “Tidak”. Sila tandakan ( / ) pada kotak untuk mewakili jawapan anda. Sila jawab semua soalan.

*The following questions require “Yes” or “No” answers. Please ( / ) in the boxes representing your answer. Please answer all questions.*

**Dalam 30 hari yang lalu, adakah anda perasan rokok elektronik diiklankan di mana-mana tempat berikut? / *In the past 30 days, have you noticed e-cigarette being advertised in any of the following places?***

| **No. / *Num.*** | **Pernyataan / *Statement*** | **Ya / *Yes*** | **Tidak / *No*** |
| --- | --- | --- | --- |
| 1. | Pada poster atau papan iklan / *On posters or billboards* |  |  |
| 2. | Dalam surat khabar atau majalah / *In newspaper or magazines* |  |  |
| 3. | Di laman web atau laman media sosial / *On websites or social media sites* |  |  |
| 4. | Di dalam radio / *On radio* |  |  |
| 5. | Di dalam televisyen / *On television* |  |  |
| 6. | Di acara seperti pameran, perayaan atau acara sukan / *At events like fairs, festivals or sporting events* |  |  |

## **Bahagian H : Ketersediaan dan Keterjangkauan**

***Part H : Availability and Affordability***

Soalan-soalan berikut memerlukan jawapan “Ya” atau “Tidak”. Sila tandakan ( / ) pada kotak untuk mewakili jawapan anda. Sila jawab semua soalan.

*The following questions require “Yes” or “No” answers. Please ( / ) in the boxes representing your answer. Please answer all questions.*

1. Adakah anda rasa sukar untuk mendapatkan rokok elektronik? / *Do you think it is difficult to get e-cigarette?*

|  | Ya / *Yes* |
| --- | --- |
|  | Tidak / *No* |

2. Adakah anda rasa harga rokok elektronik mampu milik? / *Do you think the price of e-cigarette is affordable?*

|  | Ya / *Yes* |
| --- | --- |
|  | Tidak / *No* |

## **Bahagian I : Sikap Terhadap Rokok Elektronik**

***Part I : Attitude Towards E-cigarette***

Sila tandakan ( / ) mengikut tahap persetujuan anda terhadap pernyataan di bawah mengikut skala berikut. Sila jawab semua soalan.

*Please tick ( / ) on how much you agree with the statement based on the following scale. Please answer all questions.*

| **1** | **2** | **3** | **4** | **5** |
| --- | --- | --- | --- | --- |
| Sangat tidak setuju / *Totally disagree* | Tidak setuju / *Disagree* | Tidak pasti / *Unsure* | Setuju / *Agree* | Sangat setuju / *Totally agree* |

| **No. / *Num.*** | **Pernyataan / *Statement*** | **1** | **2** | **3** | **4** | **5** |
| --- | --- | --- | --- | --- | --- | --- |
| 1. | Penggunaan rokok elektronik adalah menggembirakan / *Using e-cigarette is enjoyable* |  |  |  |  |  |
| 2. | Penggunaan rokok elektronik adalah sihat / *Using e-cigarette is healthy* |  |  |  |  |  |
| 3. | Penggunaan rokok elektronik adalah selamat / *Using e-cigarette is safe* |  |  |  |  |  |
| 4. | Penggunaan rokok elektronik adalah menyeronokkan / *Using e-cigarette is fun* |  |  |  |  |  |
| 5. | Penggunaan rokok elektronik adalah bergaya / *Using e-cigarette is stylish* |  |  |  |  |  |
| 6. | Penggunaan rokok elektronik adalah hebat / *Using e-cigarette is cool* |  |  |  |  |  |
| 7. | Penggunaan rokok elektronik adalah menarik / *Using e-cigarette is attractive* |  |  |  |  |  |

## **Bahagian J : Kesediaan Menggunakan Rokok Elektronik**

***Part J : Willingness to Use E-Cigarette***

Sila tandakan ( / ) mengikut tahap persetujuan anda terhadap pernyataan di bawah mengikut skala berikut. Sila jawab semua soalan.

*Please tick ( / ) on how much you agree with the statement based on the following scale. Please answer all questions.*

| **1** | **2** | **3** | **4** | **5** |
| --- | --- | --- | --- | --- |
| Sangat tidak setuju / *Totally disagree* | Tidak setuju / *Disagree* | Tidak pasti / *Unsure* | Setuju / *Agree* | Sangat setuju / *Totally agree* |

**Senario: Bayangkan anda berada di sebuah parti dan ramai kawan anda merokok. Salah seorang kawan rapat anda menawarkan rokok elektronik kepada anda. Apakah reaksi anda terhadap cadangan kawan anda tersebut?**

***Scenario: imagine that you are at a party and a lot of your friends are smoking. One of your intimate friends offers you e-cigarette. What is your reaction towards the proposal of your friend?***

| **No. / *Num.*** | **Pernyataan / *Statement*** | **1** | **2** | **3** | **4** | **5** |
| --- | --- | --- | --- | --- | --- | --- |
| 1. | Saya akan mengambil rokok elektronik tersebut dan menghisapnya / *I will take the e-cigarette and smoke it* |  |  |  |  |  |
| 2. | Saya akan mengatakan “Tidak, terima kasih” dan menolak cadangan kawan saya / *I will say “No, thank you” and reject my friend’s proposal* |  |  |  |  |  |
| 3. | Saya akan meninggalkan parti tersebut / *I will leave the party* |  |  |  |  |  |

## **Bahagian K : Niat Untuk Menggunakan Rokok Elektronik**

***Part K : Intention to Use E-Cigarette***

Sila tandakan ( / ) mengikut tahap persetujuan anda terhadap pernyataan di bawah mengikut skala berikut. Sila jawab semua soalan.

*Please tick ( / ) on how much you agree with the statement based on the following scale. Please answer all questions.*

| **1** | **2** | **3** | **4** | **5** |
| --- | --- | --- | --- | --- |
| Sangat tidak mungkin / *Very unlikely* | Tidak mungkin / Unl*ikely* | Tidak pasti / *Unsure* | Berkemungkinan / *Unlikely* | Berkemungkinan besar / *Very likely* |

| **No. / *Num.*** | **Pernyataan / *Statement*** | **1** | **2** | **3** | **4** | **5** |
| --- | --- | --- | --- | --- | --- | --- |
| 1. | Adakah anda fikir anda akan mencuba rokok elektronik dalam masa terdekat? / *Do you think you will try an e-cigarette soon?* |  |  |  |  |  |
| 2. | Adakah anda fikir anda akan mencuba rokok elektronik pada bila-bila masa pada tahun hadapan? / *Do you think you will try an e-cigarette anytime during the next year?* |  |  |  |  |  |
| 3. | Jika salah seorang kawan baik anda menawarkan anda rokok elektronik, adakah anda akan menggunakannya? / *If one of your best friends were to offer you an e-cigarette, would you use it?* |  |  |  |  |  |

## **Bahagian L: Tingkah Laku Berisiko Tinggi**

***Part L : High Risk Behaviour***

Soalan-soalan berikut memerlukan jawapan “Ya” atau “Tidak”. Sila tandakan ( / ) pada kotak untuk mewakili jawapan anda. Sila jawab semua soalan.

*The following questions require “Yes” or “No” answers. Please ( / ) in the boxes representing your answer. Please answer all questions.*

| **No. / *Num.*** | **Pernyataan / *Statement*** | **Ya / *Yes*** | **Tidak / *No*** |
| --- | --- | --- | --- |
| 1. | Saya pernah mengambil minuman beralkohol / *I used to drink alcohol* |  |  |
| 2. | Saya pernah mengambil bahan dadah terlarang tanpa preskripsi / *I used to take illegal drugs without a prescription* |  |  |
| 3. | Saya pernah mempunyai hubungan seks tanpa perlindungan dengan beberapa pasangan / *I used to have unprotected sex with multiple partners* |  |  |

- SOALAN TAMAT. TERIMA KASIH DI ATAS KERJASAMA ANDA -

*-END OF QUESTIONS. THANK YOU FOR COOPERATION-*
