# Supplementary material for: Prevalence and determinants of e-cigarette use among vocational college students: A cross-sectional study
Source: PLoS One. 2025 Jun 3;20(6):e0311585. doi: 10.1371/journal.pone.0311585 (PMC12132976; doi:10.1371/journal.pone.0311585)
Supplement: S4 File — (DOCX) [file pone.0311585.s004.docx]

12 Diploma courses available and all were included

Strata were formed based on the diploma programs

The number of students selected was based on the total number of students from each diploma course

Students eligibility were checked based on inclusion and exclusion criteria

Total of 700 students were invited to participate in the study

79 students did not respond, 7 students did not agree to participate

Total of 614 students participated and consented were included in the analysis

**S4 Flow diagram of study recruitment in the study**
